# P-1489. Shaken Not Stirred: Novel Antibiotic Cocktails to Combat Pan-Drug Resistant *Klebsiella pneumoniae*

**DOI:** 10.1093/ofid/ofae631.1659

**Published:** 2025-01-29

**Authors:** Jan Naseer Kaur, Albert Chen, Allison Hanna, Jack F Klem, Patricia N Holden, Liang Chen, Nicholas M Smith, Brian T Tsuji

**Affiliations:** University at Buffalo, Buffalo, New York; University of Buffalo, Williamsville, New York; University at Buffalo, Buffalo, New York; University at Buffalo, Buffalo, New York; University at Buffalo, School of Pharmacy & Pharmaceutical Sciences, Buffalo, New York; University of Buffalo, Williamsville, New York; University at Buffalo, School of Pharmacy & Pharmaceutical Sciences, Buffalo, New York; University at Buffalo, School of Pharmacy & Pharmaceutical Sciences, Buffalo, New York

## Abstract

**Background:**

*K. pneumoniae* producing Metallo-Beta-Lactamases (MBL) confers resistance to a large spectrum of beta-lactam antibiotics which is a cause for significant concern. Novel combinatorial therapies can be an effective option for treating MBL Gram-negatives. The present study assessed the synergistic potential of Polymyxin B combinatorial strategies to combat the rising tide of PDR Gram-negative strains which have emerged worldwide.

**Methods:**

*K*. *pneumoniae* producing *bla*_NDM-5_, *bla*_CTX-M-55_ and *mcr-1* was exposed to an array of therapeutic regimens in a Hollow Fiber Infection Model (HFIM) over 9 days. These regimen arms included Polymyxin B (fCss = 4 mg/L), Aztreonam (fCss = 21 mg/L) + Avibactam (fCss = 5.5 mg/L), Aztreonam (fCss = 21 mg/L) + Avibactam (fCss = 5.5 mg/L) + Polymyxin B (fCss = 4 mg/L). Bacterial plate counts were taken throughout the experiment. Samples were also fixed and imaged under a Scanning Electron Microscope.

**Results:**

Closely mirroring the growth control, Polymyxin B monotherapy did not inhibit the growth of *K. pneumoniae* S200. The clinical combination of aztreonam + avibactam demonstrated initial bactericidal activity with 6 log fold reduction (total count 1.6 log_10_ CFU/mL) over 24h followed by rapid recovery as the counts bounced back to 6.07 log_10_ CFU/mL by 48h. Reversion experiments demonstrated complete regrowth to 9.53 log_10_ CFU/mL by the 216h time point. In contrast, the addition of polymyxin B to the β-lactam/β-lactamase inhibitor combination resulted in prolonged marked bactericidal activity with 6.05 log fold reduction within 24h and eventually below the detectable limits over 168h.This was supported by tracking the evolution of salient phenotypic alterations using SEMs over 216h that showed filamentation in response to aztreonam containing regimens and lack of viable cells as well as overwhelming presence of cellular debris in Polymyxin B combination arm at 24 and 168h.

**Conclusion:**

The results of the present study suggest that despite the intrinsic *mcr-1* and *bla*_NDM-5_ resistance mechanisms our novel antibiotic cocktails show significant promise. Novel therapeutic options that enhance combinatorial pharmacodynamics are necessary to combat the urgent threat of MBL Gram-negative strains which are resistant to all antibiotics.

**Disclosures:**

**All Authors**: No reported disclosures

